# Study on the Variation Law of the Main Mechanical Properties in the Processing of Longjing Tea

**DOI:** 10.3390/foods12132587

**Published:** 2023-07-03

**Authors:** Chen Li, Xiaoyong Wang, De Zhang, Yuqiong Chen, Xinfeng Jiang, Dejiang Ni

**Affiliations:** 1 Jiangxi Institute of Economic Crops, Nanchang 330202, China; hanwuji1110@126.com; 2 College of Horticulture and Forestry Sciences, Huazhong Agricultural University, Wuhan 430070, China; xiaoyongw@webmail.hzau.edu.cn (X.W.); zdybfq@163.com (D.Z.); chenyq@mail.hzau.edu.cn (Y.C.)

**Keywords:** Longjing tea, processing, tensile properties, texture properties, water content

## Abstract

Fresh tea leaves, both single bud and one leaf with a bud, were used as the test materials in this study. The variation in the main mechanical properties, such as texture and tensile properties, during the processing of Longjing tea was examined by using texture profile analysis (TPA) and stress–strain tensile tests. The plasticity showed a trend of first increasing and then decreasing during the processing, whereas the elasticity displayed the opposite tendency. The flexibility reached a maximum during the fixing stage and then slowly declined with a relatively small change. Initially, the maximum force dropped down and then gradually elevated later.Both the tensile strength and the fracture strain indicated an upward movement at the beginning and then a downfall afterward. The elastic modulus changed little before the final panning stage, then raised significantly. The correlation analysis revealed that the flexibility of tea leaves was highly positively correlated with water content. At water content of 30% and 50%, the plasticity and flexibility of tea leaves reached a clear peak and the maximum force was at a low level, which is suitable for the shaping of Longjing tea. The results also demonstrated that the main mechanical properties of different tender materials change differently during the processing. The research findings can provide parameters for optimizing the mechanical design and processing technology of Longjing tea.

## 1. Introduction

West Lake Longjing Tea is produced in the West Lake District of Hangzhou, Zhejiang Province, China, and is renowned for its “green color, fragrant aroma, sweet taste, and beautiful appearance” both domestically and abroad [1]. Traditional Longjing tea is completely hand-crafted and involves ten techniques for frying the tea leaves, including shaking, tapping, expanding, squeezing, flinging, clutching, pushing, buttoning, pressing, and grinding [2]. Hand-crafted Longjing tea has a flat, smooth, and moderate-width appearance with a strong and refreshing aroma along with a rich and mellow taste that meets the quality characteristics of traditional Longjing tea [3]. However, this process requires highly skilled workers and has lower efficiency, high labor intensity, and unstable product quality, making it difficult to achieve large-scale and standardized production. As tea leaves are fragile, collisions and friction can easily damage them during mechanical processing, affecting not only the subsequent processing method but also the color and surface of tea leaves, which in turn affects their commercial value. The existing mechanical equipment still requires high operational skills and has unstable tea quality. Further improvement and optimization of continuous automated production machinery are needed.

Common mechanical testing methods for crops include tensile compression, bending, and shearing. Currently, there are many studies on the mechanical properties of wheat, corn, and barley stalks [4], and the mechanical properties of stalks tensile tests can provide a reference for positioning, supporting, and clamping materials during harvest and processing [5]. The bending strength, elastic modulus, and moment of inertia of stem materials have a similar impact on the lodging index, providing a basis for evaluating the lodging resistance of crops [6]. Crops harvesting and vegetable picking are usually accomplished by stem shearing and the stem shearing properties have a direct impact on the efficiency of these tasks [7]. The design of harvesting and picking machinery should consider reducing fruit damage [8]. Additionally, in the process of fruit transportation, damage, and stress are directly related, and their relaxation parameters are also associated with storage and quality [9,10]. Therefore, the study of the mechanical properties of agricultural products is significant for the entire process of harvesting, processing, transportation, storage, and quality evaluation.

The physical properties of tea include friction, mechanical properties, and flow properties [11]. The friction and flow characteristics of tea leaves are mainly used for the rational configuration of tea machines during deep processing. During the sieving process, the movement law of tea leaves is related to the inclination angle and friction coefficient of the screen surface [12] There is a significant difference in friction between different grades of crude tea and tea machines [13]. The selection of sieving methods such as round sieves, shaking sieves, and floating sieves should consider the material characteristics of tea leaves [14]. Tea picking is seasonal and requires intense labor. Therefore, mechanized harvesting is an inevitable trend [15,16,17]. Research on the mechanical properties of tea can provide a theoretical basis for the development of tea-picking machines with good picking efficiency and high work efficiency. The current blade-cutting method used in tea-picking robots does not meet the requirements for harvesting high-quality green tea. Designing bionic picking finger structures [18] and pinch-cut combined picking devices [19] based on the mechanical and morphological properties of tea leaves can provide valuable references for reducing damage during the picking of tender tea leaves. The basic physical property changes of tea can provide relevant basic data and prediction models for the tea processing process [20], different processing techniques are established based on the tenderness, water content, and mechanical properties of leaves, which can fully tap into the value of fresh leaves and promote the shape of tea leaves. The variation of mechanical properties of rolled leaves in oolong tea bags was studied. It provided a theoretical scientific basis for improving the rolling technology of oolong tea [21]. Previously, we studied the change of physical properties in needle-shaped green tea processing and found that plasticity was higher during the rolling and shaping stage (water content of 23% to 58%) and the trend of elasticity was opposite to that of plasticity [22]. The changes in elasticity and plasticity may be related to substances such as pectin [23,24]. It has been shownthat the mechanical properties of tea are also correlated with its quality characteristics. There are significant differences in the biochemical composition and aroma quality of Oolong tea under different shaping force modes [25], and the elasticity index of particle-shaped green teais negatively correlated with the shape and color quality, while the plasticity index is only negatively correlated with the color quality [26].

Currently, the research on the texture properties and tensile properties related to the flat and smooth appearance quality characteristics of Longjing tea during processing has not been investigated. In this paper, a single bud and one bud with one leaf of “Fudingdahao“ were selected as the research objects. The main mechanical properties such astexture and tensile properties during Longjing tea processing were tested and the changes intensile strength, elastic modulus, and other mechanical indexes of tea with different tenderness during processing were analyzed. The study providesa parameter basis for the design optimization of Longjing tea processing machinery, reduces the broken tea rate during the mechanical processing of flat-shaped tea, and also providesbasic information for the automation and intelligence of tea processing.

## 2. Materials and Methods

### 2.1. Materials

The tea plant variety was “Fudingdahao” of *Camellia sinensis* cv. The fresh leaves with sing budand one bud with one leaf were picked on April (sunny day) from the South Lake Tea Plantation Base of Huazhong Agricultural University (Wuhan, China).

### 2.2. Instruments and Equipment

Continuous roller fixation machine (6CST-50, Zhejiang Green Peak Machinery Co., Ltd., Quzhou, China), Roasting machine for flat tea (6CCB-80E, Zhejiang Green Peak Machinery Co., Ltd., Quzhou, China), Fixing machine (6CZG-60, Zhejiang Green Peak Machinery Co., Ltd.), Electric thermostatic blast drying oven (DHG-9246, Shanghai Jinghong Experimental Equipment Co., Ltd., Shanghai, China), Texture analyzer (TMS-PRO, Food Technology Corporation in USA), UTM2000 Electronic universal testing machine (Shenzhen Suns Technology Co., Ltd., Shenzhen, China).

### 2.3. Tea Processing

**Figure 1:**
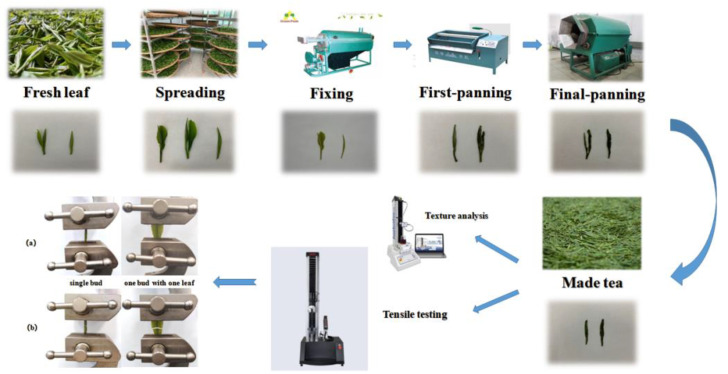
The Longjing tea manufacturing flow chart and the determination of texture and tensile properties. The test state of the sample during tensile testing is shown in (**a**). When the stress reached the maximum value, the sample broke within the standard distance, as shown in (**b**).

The Longjing tea processing was achieved as follows: fresh tea leaves were plucked, spread, fixed, firstpanned and finalpanned, as shown in Figure 1. After entering the factory, the fresh leaves were naturally spread indoors at a temperature of 26 °C and a relative humidity of 55%, with a leaf thickness of around 3 cm, a spreading time of 4 h, and a water content of about 70%. The 6CST-50 type continuous roller fixation machine was used for fixing. The air temperature was 120 °C–140 °C at a distance of 30 cm from the inlet and the bottom of the pot, and the water content of the fixing leaves was about 60%. The fixing leaves were shaped in a 6CCB-80 roasting machine for flat tea after 30–60 min of rejuvenation. The temperature in the bottom of the pot was 150–180 °C, the amount of leaves was 1.5 kg, and the water content was kept at 20 ± 2%. The six-edge roasting machine was used for final panning, with a temperature of 80–100 °C, a time of 8–10 min, and a water content of 10 ± 2%.

### 2.4. Sampling

By using the simple random sampling method [27], the fresh leaves were sampled after entering the factory and the samples were also taken after the spreading and fixing process. Two sets of samples were taken in the first-panning process and three sets were taken in the final-panning process. The water content and mechanical properties were tested immediately after full rejuvenation, with a testing time not exceeding 1 h.

### 2.5. Water Content Measurement [28]

The water content of tea leaves during the Longjing tea processing was measured using the national standard GB/T8304-2013 “Tea Determination of moisture content” with three repetitions per sample.

### 2.6. Texture Properties Measurement [29]

**Figure 2:**
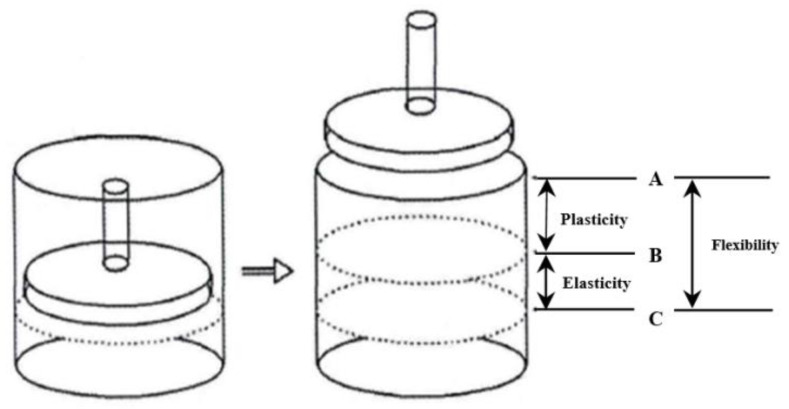
Plasticity, elasticity, and flexibility determination schemes.

The TMS-PRO texture analyzer was used. The sample was placed in a transparent cylindrical container (with a diameter of 80 mm and a height of 85 mm) without vibration or compaction and was slightly shaken so that the surface of the tea leaves was level with the edge of the container. The TPA test was performed with a 38.1 mm cylindrical extrusion probe. As shown in Figure 2, at first, the sample was pressed to point A with a force of 0.3 N to measure the original height of the sample. Next, the sample was pressed to point C with a force of 4.9 N to measure the lowest height of the sample. After the probe returned to the starting position for 60 s, the sample was pressed to point B with a force of 0.3 N to measure the recovery height of the sample, and each sample was repeated three times.

### 2.7. Tensile Properties Measurement [30]

The UTM2000 electronic universal testing machine was used, with a maximum load of 100 N, and a speed range of 0.001–500 mm/min. The 100 N sensor and customized tensile clamp (integrated clamp-sensor, with treated clamp surface to prevent tea leaf breakage) were selected to measure the tensile properties of tea leaves during processing. The data was collected through the force sensor during the tensile test, and the testing machine was equipped with Material Test 4.0 software, which can dynamically display the force, deformation, displacement, loading speed, and change curve in real-time. The tea sample was clamped between the upper and lower clamps of the testing machine, and the sample was placed in the middle of the clamp, perpendicular to the horizontal plane. The initial force was 1 N, and the machine operated slowly (with a speed of 20 mm/min) until the sample broke. The test state of the sample during tensile testing is shown in Figure 1a. When the stress reached the maximum value, the sample brokewithin the standard distance, as shown in Figure 1b. Twenty tea sticks were randomly selected from each sample for measurement (leaves were selected for the one bud with one leaf of raw material). The elastic modulus was calculated based on the load-displacement curve using Equation (1).

(1) $$E=\frac{Fl}{\Delta lA}$$

where *E* is the elastic modulus, GPa; *F* is the tensile force, N; *l* is the length of the tea sample, m; Δl is the Deformation of the tea sample, m; *A* is the cross-section area of the tea sample, the product of the tea sample’s width and thickness, m^2^.

The tensile strength was calculated by Equation (2).

(2) $$\sigma =\frac{F_{\max}}{A}$$

where σ is the tensile strength, MPa; $F_{\max}$ is the maximum force, N.

## 3. Results and Discussion

### 3.1. Changes in Water Content of Longjing Tea during Processing

Water content is a main factor affecting the changes in the physical and chemical properties of tea during processing. The water content of Longjing tea gradually decreased during the processing (Table 1). The trend of water content changes in tea during processing was consistent among different raw materials.

### 3.2. Changes in Texture Properties of Longjing Tea during Processing

The texture properties of tea mainly include flexibility, plasticity, and elasticity. Flexibility refers to the ease of deformation, plasticity refers to the ability to undergo plastic deformation or permanent deformation, and elasticity refers to the ability to undergo elastic deformation or recover from deformation. In the shaping process of tea, the three properties need to be combined reasonably to achieve better deformation under external forces [11].

**Figure 3:**
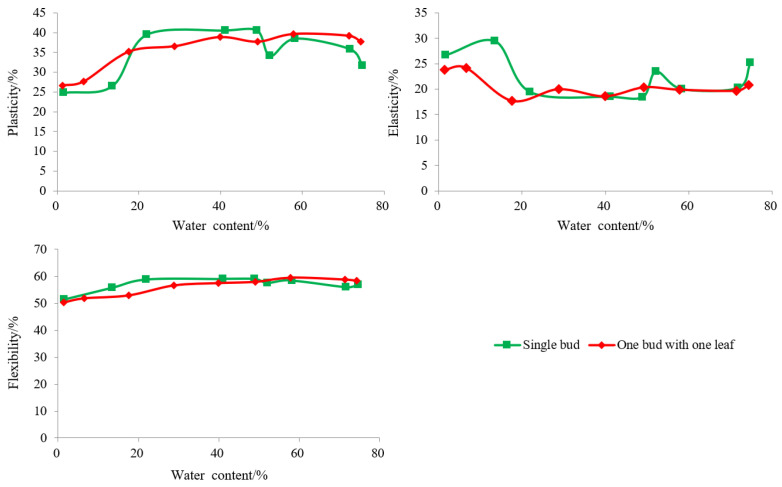
The changing pattern of texture properties in Longjing tea processing.

As shown in Table 2, the plasticity of Longjing tea during processing first increased and then decreased, while the elasticity first decreased and then increased. This is because the initial water content of Longjing tea was high during processing, and the pores between tea cells were small, so the possibility of material movement inside the cells was low. With the decrease in water content, the pores between cells began to increase, and the interaction between leaf cells decreased, resulting in an increase in plasticity and a decrease in elasticity. With the further decrease in water content, the internal structural interaction of the leaf increased as a result the internal force also increased, causing a decrease in plasticity and an increase in elasticity. The plasticity changes in Longjing tea during processing were consistent for different raw materials at different levels of tenderness. Single bud raw material reached the maximum plasticity value (40.5%) in the final-panning process, while one bud with one leaf raw material reached the maximum plasticity value (39.7%) after the fixing process. This was because the water content of single bud raw material was at a relatively high level during the final-panning process and the internal force of the leaf was relatively small at this time. One bud with one leaf of raw materials had a relatively high water content during the fixing process. The plastic of one bud with one leaf of raw material significantly decreased in the late stage of finalpanning and drying. The plasticity value of tea remained at a relatively high level (water content 50%–20%) Figure 3. The plasticity value of one bud with one leaf (37.7%) was significantly higher than that of a single bud (31.7%). However, in the final-panning process, the plasticity of a single bud was slightly higher than that of one bud with one leaf. The elasticity of Longjing tea was at the lowest level during the early stage of firstpanning (water content 49%–52%). The difference in elasticity between one bud with one leaf (20.8%) and single-bud tea (25.1%) was significant. As processing progresses, the discrepancies were reduced, but the elasticity value of a single bud was still significantly higher than that of one bud with one leaf in the late stage of finalpanning and drying. The overall change in flexibility of Longjing tea during processing was not significant for different levels of tenderness Figure 3.

### 3.3. Changes in Tensile Properties of Longjing Tea during Processing

**Figure 4:**
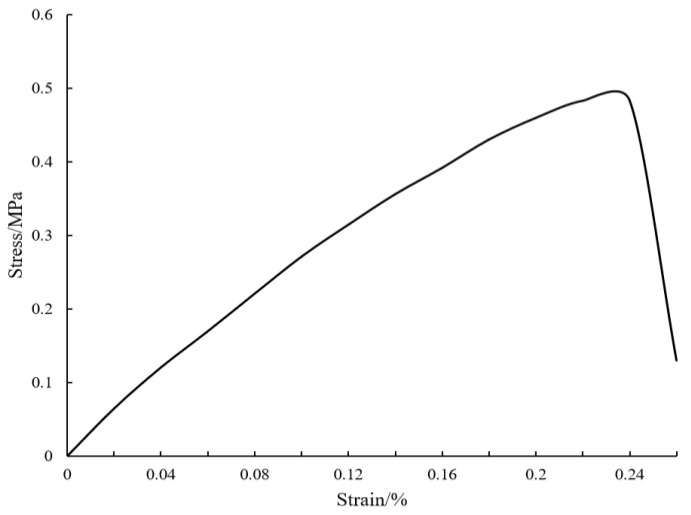
The stress and strain of tea leaves.

The tensile process of tea leaves goes through three stages [11] (Figure 4). The first is the stage of elastic deformation. The stress and strain show an approximately linear relationship, and the deformation of tea leaves is almost recoverable. This stage follows Hooke’s law, and the ratio of stress to strain is the elastic modulus. The second is the stage of plastic deformation, which continues to stretch to the allowable stress of leaves to maintain the integrity of the cell structure, which is the maximum stress point. At this stage, the stress and strain are non-linear related. The greater the strain, the better the flexibility of the tea leaves. The third is the fracture stage. When the plastic deformation of tea leaves reaches a certain degree, continued stretching will cause a fracture, and the corresponding stress is the fracture stress whereas the corresponding strain is the fracture strain.

**Figure 5:**
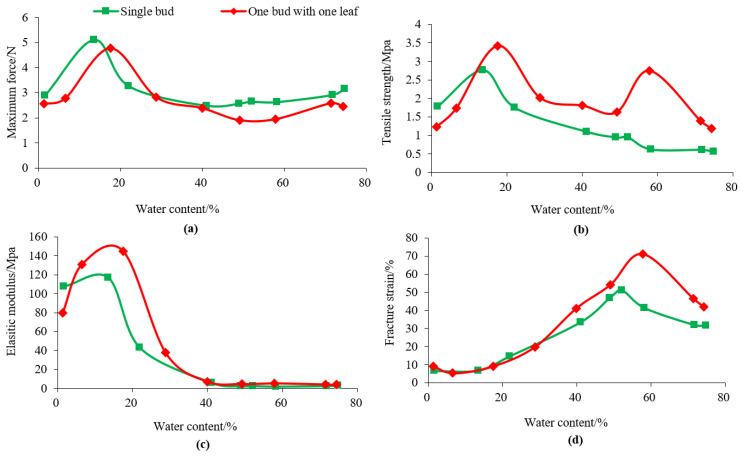
The changing pattern of maximum force (**a**), tensile strength (**b**), elastic modulus (**c**) and fracturr strain (**d**) in Longjing tea processing.

The maximum force refers to the maximum stress that can be sustained by the tea leaves during the stretching process. In the processing of Longjing tea made from different tender raw materials, the maximum force of the tea leaves showed a trend of first increasing and then increasing. As shown in Figure 5a, throughout the processing procedure, the maximum force of the tea leaves gradually increased with the decrease in water content and then gradually decreased with the decrease of water content. This may be due to the different magnitudes of plasticity deformation and the elasticity deformation of tea leaves during the processing as water content decreases. As the water content decreased, the plasticity of tea leaves decreased while the elasticity increased. However, the magnitude of the decrease in plasticity was greater than the increase in elasticity, causing the stretching process to shift from being dominated by plastic deformation to being dominated by elastic deformation. The maximum level was reached after the water content decreases to below 20% in the finalpanning process (Figure 5a). It was likely due to the tighter structure of the tea leaves internal tissue during the water loss, resulting in an increase in the maximum force. Since the water content of the finished tea was low and the tea strips were brittle, the maximum force of the tea leaves dropped sharply after the drying process. The maximum force of the single bud raw material was greater than that of the one bud with one leaf. Compared with the single bud, the leaf of the one bud with one leaf was thinner, and thus could resist a relatively smaller maximum force during the tensile testing.

Tensile strength refers to the force that produces the maximum uniform plastic deformation of tea leaves and reflects the material’s ability to resist destruction. As Formula (2) shows, tensile strength was not only related to the maximum force but also to the cross-sectional area of the tea sample. During the processing of Longjing tea, the decrease in water content caused the leave to gradually shrink, resulting in changes in the cross-sectional area. Before the first-panning process, the maximum force of a single bud was greater than that of one bud with one leaf, but the tensile strength was smaller than that of one bud with one leaf, indicating that the maximum force dominated before the first-panning process, while the cross-sectional area of the tea sample dominated during the final-panning and drying process. According to Table 3, during the processing of single buds, the tensile strength increased slowly in the early stage but increased significantly during the final-panning process. The tensile strength of one bud with one leaf increased briefly during the fixing process due to the loss of water and the cross-sectional area decreasing sharply. The tensile strength of the one bud with one leaf before the second finalpanning process was greater than that of the single bud, while the third finalpanning and drying stage showed the opposite result. This was because the maximum force of the single bud during the third finalpanning process was close to twice that of the one bud with one leaf. The tensile strength of the single bud during the drying process was higher than that of the one bud with one leaf, partly because the water content of the two was closer at this time, while the one bud with one leaf was thicker and had a larger cross-sectional area. The changes from spreading to fixing were slightly different, with the tensile strength of the one bud with one leaf increasing from 1.40 MPa to 2.76 MPa, while the tensile strength of the single bud barely increased during this process. The difference in the changing trend between the single bud and the one bud with one leaf was most evident after the fixing process (Figure 5).

The elastic modulus is the ratio of stress to strain within the elastic range [11], which measures the size of an object’s ability to resist elastic deformation [22]. Comparatively, it can be seen that during the processing of Longjing tea, the elastic modulus values of the one bud with one leaf were higher than that of the single bud before the final panning process. This indicated that the one bud with one leaf had a strong ability to resist deformation than the single bud. This was because one bud with one leaf resisted external forces together with the veins and the mesophyll, while the single bud only relied on the mesophyll to resist the external forces, and the veins were relatively fewer, so the mesophyll generally split first and then the veins resisted external force. The change in elastic modulus was not significant until the water content drops to 40% (Figure 5c). Afterward, as the tea leaves loosed more water, the elastic modulus increased significantly. In the later stage of finalpanning (water content 15%–17%), the elastic modulus of the leaves and buds reached 145 MPa and 117 MPa, respectively, and both decreased during the drying process.

The fracture strain refers to the strain that occurs when the tea leaves are stretched to the maximum and fracture, reflecting the plastic capacity of the tea leaves [22]. During the processing, as the water content decreased, the fracture strain showed an increasing-then-decreasing trend. The reason might be that the tea leaves were relatively intact in the early stage of processing, but with the increase in friction between the tea leaves and the processing equipment in the later stage, the number of collisions between tea leaves increased, and micro-cracks appeared on the surface of the tea leaves, causing stress concentration and a decrease in fracture strain. The inflection point appeared in the fixing process (water content 58%). The change trend of different tender raw materials was consistent and the strain of the one bud with one leaf was significantly higher than that of the single bud before the fixing process. With the processing, the difference between the two gradually decreased, and there was almost no difference in the later stage of processing.

### 3.4. Correlation Analysis

**Figure 6:**
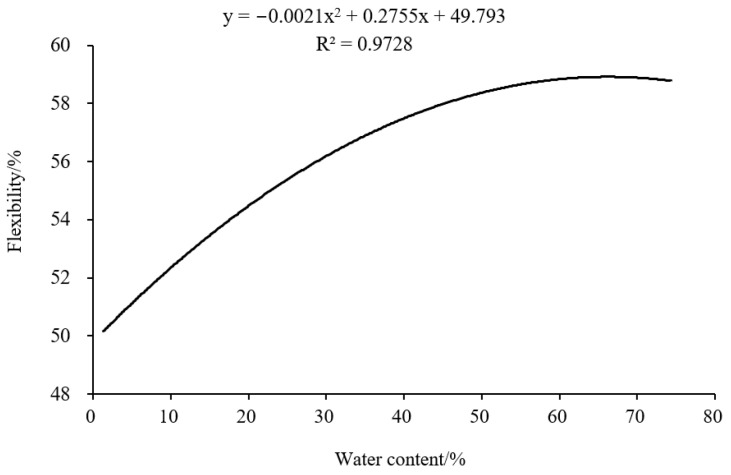
The fitted curve of flexibility and water of tea leaves in Longjing tea processing (one bud with one leaf).

The flexibility of one bud with one leaf of raw material was highly significant and positively correlated with the water content, according to the correlation analysis of various parameters in Longjing tea processing (Table 4 and Table 5). The relationship between the flexibility and water content of tea leaves with one bud with one leaf was obtained by SPSS fitting: y = −0.0021x^2^ + 0.2755x + 49.793, with a coefficient of determination R^2^ = 0.9728. The fitted curve was shown in Figure 6. The correlation of each parameter index was significantly different in the process of raw materials with different tenderness. The plasticity of single-bud raw material was highly significant and negatively correlated with the elasticity, and the tensile strength was highly significant and positively correlated with the elastic modulus. The plasticity of one bud with one leaf of raw material was highly significant and positively correlated with the flexibility.

## 4. Discussion

During the processing of Longjing tea, the texture properties and tensile properties of the tea leaves changed with variations in water content and tea leaf shape. The tea leaf shape gradually shrunk under the combined effect of heat and mechanical force, eventually forming the smooth and flat unique shape of Longjing tea. Water content is often used as an important quantitative indicator to judge the beginning and the moderate completion of each process in tea production [31]. The fresh tea leaves had high water content and poor plasticity and flexibility. After being spread out, the tea leaves lost water slowly, causing them to lose luster, became withered, the cell space expanded, the repulsive force between internal contact of the leaves reduced, the elasticity decreased, and the flexibility and plasticity increased [11]. The fracture strain increased, and the maximum force decreased. The water content of the tea leaves dropped to about 58% after high-temperature fixing. Tea leaves were subjected to centrifugal force and friction force in the cylinder, the tea stick shrunk, the cohesiveness dropped and the plasticity and flexibility further increased [32] whereas the maximum force further decreased. During the shaping process, the tea leaves were subject to the force of the roasting machine, including the forces from the frying leaf plate and the pot surface, as well as frictional forces and extrusion forces between the tea leaves. This resulted in a smooth and flat shape, and the large loss of water caused the tea strips to become tightly bound, increasing their tensile strength. The plasticity values of the tea leaves remained at a high level during this process. The tea strips gradually became flattened during the shaping process, and the cross-sectional area under stress gradually decreased. As the water content and external forces decreased, the tea strips became tighter, increasing internal stress and tensile strength [33,34]. During the final-panning procedure, the tea leaves lost water due to heat, and under the convex ribs on the inner wall of the solidification machine cylinder, friction and extrusion increased, causing the strips to become even tighter, achieving the goal of solidification and drying at the same time [35]. Plasticity and flexibility decreased significantly at this point, while elasticity increased. The tea leaves were subjected to heat during the drying process and the water content dropped to below 5%. The tea strips became hard and brittle, causing an increase in strength, a decrease in elasticity, and a decrease in plasticity, flexibility, and strain indicators.

The mechanical properties of tea are an important branch of agricultural materials engineering and are also an important basis for innovation in tea shaping equipment [14]. Studying the texture properties and tensile properties of tea during processing is not only beneficial to improving processing technology, enhancing processing level, and stabilizing tea quality but also has important significance for the improvement and development of processing machinery and equipment. According to the different mechanical properties of different fresh leaves with different tenderness, the design of automated conveying devices and the roasting method of Longjing tea should be different from existing equipment. The plasticity and flexibility of one bud with one leaf raw materials were both greater than those of single bud whereas the elasticity was lower than that of single bud raw materials. Therefore, attention should be paid to the difference between raw materials during the transportation and stacking of fresh leaves, and the thickness of the single bud transmission and stacking should be controlled to be less than that of one bud with one leaf. Different heights of conveyor belts with teeth can be set up to adjust the distance between the uniform leaf regulator and the belt, thereby reasonably controlling the fixing degree of different raw materials. In the shaping process of Longjing tea, compression, vibration, and friction comprehensive force are the main forces. Through the reciprocating motion of the roasting pot and the pressure of the roasting-leaf device, the tea leaves collided and pressed to become flat and smooth. In the design of the Longjing tea frying method and program, during the first-panning processing, the tea leaves moved along the axial sequence with the plate on the cam track, and the elasticity of the single bud in the early stage (water content 49%–52%) was obviously higher than that of leaves. At this time, different leaf weights or pressure treatments could be appropriately set to facilitate the shaping of tea strips with different raw materials. After spreading and cooling back to the dampness of the tea leaves, before entering the final-panning process, the elasticity of tea leaves obviously increased at this stage (water content 20%–40%). By combining the leaves of the firstpanning stage together, increasing the leaf weights, and relying on the interaction force between the tea leaves and tea strips, the shape can be further fixed [35]. In the early stage of finalpanning, the plasticity of single-bud raw materials was greater than that of one bud with one leaf, and the pressure of the plate could be moderately increased when stir-frying single bud; In the later stage of finalpanning and the drying processing, tea strips were further fixed under the effect of humidity and heat. The water was lost quickly and the tea strips became hard and brittle. At this time, the plasticity of different raw materials was low, and they could not be fried again with increased pressure; otherwise, it would cause breakage and reduce the rate of regular tea. Especially for one bud with one leaf of raw materials, the flexibility and plasticity decreased quickly in the later stage, and the tea strips were more likely to be broken under stress and the temperature should not be too high at this time.

## 5. Conclusions

Measuring the mechanical properties of tea processing is essential for process optimization and machine design. In this study, tea samples with different tenderness and processing stages were tested for texture and tensile properties. The results showed that during the processing of Longjing tea, as the water content decreased, the plasticity and flexibility of tea increased first and then decreased, while the elasticity decreased first and then increased. The maximum force showed a decreasing and then increasing trend, reaching its maximum value during the final-panning process. Tensile strength and fracture strain showed an increasing-then-decreasing trend, with the maximum tensile strength appearing during the final-panning process and the maximum fracture strain appearing after fixing. The initial change of elastic modulus was not obvious, but a sudden increase was observed in the later stage of the final-panning process (water content 15%–17%), with the leaf and bud reaching 145 MPa and 117 MPa respectively. When the water content was between 30% and 50%, the plasticity and flexibility of tea leaves reached a clear peak, the maximum force was at a low level, and the fracture strain was at a high level, which is suitable for the shaping of Longjing tea. The change in texture properties and tensile properties of raw materials with different tenderness during processing was not the same. The study provides valuable insights into the mechanical property changes in the processing of Longjing tea, facilitating the development of intelligent processing machinery and optimization of processing techniques to enhance and stabilize tea product quality. This method can also be applied to other types of tea. In further research, the interaction between moisture content, leaf temperature, and applied force during tea shaping can be investigated from thermal and mechanical perspectives. In addition, different tea tree varieties exhibit variations in leaf morphology and leaf quality. Thus, further research is needed to explore the main mechanical property differences and their correlation with tea tree adaptability for fresh leaves, intermediate products, and finished tea materials.

## Figures and Tables

**Table 1:** The change of water content of tea leaves in Longjing tea processing (%).

Picking Standard	Fresh Tea Leaves	Spreading	Fixing	First Panning 1	First Panning 2	Final Panning 1	Final Panning 2	Final Panning 3	Drying
I	74.8 ± 0.2	71.8 ± 0.3	58.3 ± 1.8	52.1 ± 0.2	48.9 ± 0.1	42.1 ± 0.6	22.0 ± 1.6	13.5 ± 0.4	1.6 ± 0.2
II	74.3 ± 0.3	71.4 ± 0.8	57.8 ± 3.3	49.1 ± 0.9	40.0 ± 1.1	28.8 ± 0.5	17.5 ± 0.3	6.55 ± 0.1	1.3 ± 0.1

Note: I: Single bud; II: one bud with one leaf.

**Table 2:** The change of texture properties of tea leaves in Longjing tea processing (%).

Texture Properties	Picking Standard	Fresh Tea Leaves	Spreading	Fixing	First Panning 1	First Panning 2	Final Panning 1	Final Panning 2	Final Panning 3	Drying
Plasticity	I	31.6 d	35.7 bc	38.4 ab	34.1 cd	40.5 a	40.5 a	39.4 a	26.4 e	24.8 e
	II	37.7 ab	39.2 a	39.7 a	37.7 ab	38.8 ab	36.5 ab	35.1 b	27.6 c	26.6 c
Elasticity	I	25.1 bc	20.3 d	20.0 d	23.5 c	18.4 d	18.5 d	19.4 d	29.4 a	26.7 ab
	II	20.8 ab	19.7 c	19.9 c	20.4 bc	18.6 c	20.0 c	17.7 c	24.2 a	23.8 ab
Flexibility	I	56.8 bc	56.0 c	58.5 ab	57.6 ab	58.9 a	59.0 a	58.9 a	55.8 c	51.5 d
	II	57.7 a	58.8 a	59.5 a	58.0 a	57.4 a	56.5 a	52.8 b	51.8 b	50.4 b

Note: I: Single bud; II: one bud with one leaf; different lowercase letters in the same column indicate significant differences at *p* < 0.05.

**Table 3:** The change of tensile properties of tea leaves in Longjing tea processing (%).

Texture Properties	Picking Standard	Fresh Tea Leaves	Spreading	Fixing	First Panning 1	First Panning 2	Final Panning 1	Final Panning 2	Final Panning 3	Drying
Maximum Force	I	3.15 bc	2.91 bcd	2.62 cd	2.64 cd	2.56 cd	2.48 d	3.26 b	5.11 a	2.89 bcd
	II	2.44 b	2.57 b	1.95 b	1.91 b	2.39 b	2.82 b	4.77 a	2.78 b	2.56 b
Tensile Strength	I	0.57 d	0.61 d	0.62 d	0.95 c	0.95 c	1.10 c	1.75 b	2.76 a	1.78 b
	II	1.18 d	1.40 cd	2.76 b	1.63 cd	1.81 cd	2.02 c	3.42 a	1.74 cd	1.24 d
Elastic Modulus	I	3.11 d	2.72 d	2.17 d	2.55 d	2.42 d	5.77 d	43.3 c	117 a	107 b
	II	4.12 d	4.12 d	5.19 d	4.59 d	7.23 d	37.8 c	145 a	131 a	79.8 b
Fracture Strain	I	31.6 c	31.8 c	41.2 b	51.0 a	47.0 a	33.6 c	14.5 d	6.79 e	6.57 e
	II	42.0 c	46.7 bc	71.3 a	54.0 b	41.2 c	19.8 d	9.01 e	5.34 e	9.22 e

Note: I: Single bud; II: One bud with one leaf; Different lowercase letters in the same column indicate significant differences at *p* < 0.05.

**Table 4:** Correlation analysis of various parameters of Longjing tea (single bud) (%).

Correlation Coefficient	Water Content	Plasticity	Elasticity	Flexibility	Maximum Force	Tensile Strength	Elastic Modulus	
Water Content	1							
Plasticity	0.465	1						
Elasticity	−0.418	−0.958 **	1					
Flexibility	0.453	0.881 **	−0.707 *	1				
Maximum Force	−0.431	−0.583 *	0.717 *	−0.252	1			
Tensile Strength	−0.876 **	−0.541	0.596	−0.349	0.797	1		
Elastic Modulus	−0.873 **	−0.780	0.749 *	−0.685	0.717	0.909 **	1	
Fracture Strain	0.749 *	0.632	−0.575 *	0.606	−0.661	−0.790 *	−0.884 *	1

Note: ** indicates a significant difference at *p* < 0.01; * indicates a significant difference at *p* < 0.05.

**Table 5:** Correlation analysis of various parameters of Longjing tea (one budwithoneleaf).

Correlation Coefficient	Water Content	Plasticity	Elasticity	Flexibility	Maximum Force	Tensile Strength	Elastic Modulus	
Water Content	1							
Plasticity	0.837 **	1						
Elasticity	−0.450	−0.817 **	1					
Flexibility	0.927 **	0.930 **	−0.548	1				
Maximum Force	−0.434	−0.208	−0.331	−0.513	1			
Tensile Strength	−0.201	0.207	−0.565	−0.061	0.619	1		
Elastic Modulus	−0.816 **	−0.725 *	0.279	−0.875 **	0.765 *	0.423	1	
Fracture Strain	0.829 **	0.793 *	−0.393	0.901 **	−0.651	−0.034	−0.850 **	1

Note: ** indicates a significant difference at *p*< 0.01; * indicates a significant difference at *p* < 0.05.

## Data Availability

Data available in a publicly accessible repository. Data is contained within the article.
